# The Potential of Pathological Protein Fragmentation in Blood-Based Biomarker Development for Dementia – With Emphasis on Alzheimer’s Disease

**DOI:** 10.3389/fneur.2015.00090

**Published:** 2015-05-11

**Authors:** Dilek Inekci, Ditte Svendsen Jonesco, Sophie Kennard, Morten Asser Karsdal, Kim Henriksen

**Affiliations:** ^1^Nordic Bioscience, Biomarkers and Research, Herlev, Denmark; ^2^Systems Biology, Technical University of Denmark, Lyngby, Denmark

**Keywords:** dementia, Alzheimer’s disease, biomarkers, blood, post-translational modifications

## Abstract

The diagnosis of dementia is challenging and early stages are rarely detected limiting the possibilities for early intervention. Another challenge is the overlap in the clinical features across the different dementia types leading to difficulties in the differential diagnosis. Identifying biomarkers that can detect the pre-dementia stage and allow differential diagnosis could provide an opportunity for timely and optimal intervention strategies. Also, such biomarkers could help in selection and inclusion of the right patients in clinical trials of both Alzheimer’s disease and other dementia treatment candidates. The cerebrospinal fluid (CSF) has been the most investigated source of biomarkers and several candidate proteins have been identified. However, looking solely at protein levels is too simplistic to provide enough detailed information to differentiate between dementias, as there is a significant crossover between the proteins involved in the different types of dementia. Additionally, CSF sampling makes these biomarkers challenging for presymptomatic identification. We need to focus on disease-specific protein fragmentation to find a fragment pattern unique for each separate dementia type – a form of protein fragmentology. Targeting protein fragments generated by disease-specific combinations of proteins and proteases opposed to detecting the intact protein could reduce the overlap between diagnostic groups as the extent of processing as well as which proteins and proteases constitute the major hallmark of each dementia type differ. In addition, the fragments could be detectable in blood as they may be able to cross the blood–brain barrier due to their smaller size. In this review, the potential of the fragment-based biomarker discovery for dementia diagnosis and prognosis is discussed, especially highlighting how the knowledge from CSF protein biomarkers can be used to guide blood-based biomarker development.

## Introduction

Dementias are brain disorders that cause a progressive decline in mental function. In 2009, it was estimated that 35.6 million people were suffering from dementia worldwide and this number is expected to be 65.7 million by 2030 and 115.4 by 2050 (1). Alzheimer’s disease (AD) is the most common cause of dementia, and accounts for 60–70% of all cases. Other common causes of dementia are dementia with Lewy bodies (DLB), vascular dementia (VaD), frontotemporal lobar degeneration (FTLD), and corticobasal degeneration (CBD). In addition to this, mixed dementias are also commonly seen (2–4).

The major risk factor for developing dementia is age, with increasing prevalence after age 65, followed by family history, environmental factors, and mutations (4). Cognitive and neuropsychiatric symptoms are the key clinical features of dementia (5).

The diagnosis of dementia is challenging and early and moderate stages of dementia are rarely detected thereby limiting the potential for early intervention. Additionally, a high number of dementia cases are left without a diagnosis (6).

It is generally accepted that there is a need for early diagnosis of dementia and many efforts have been made to develop early biomarkers with the ability to identify the pre-dementia stage of the disease before the onset of cognitive decline and brain degeneration (7, 8).

Another challenge is the differential diagnosis of dementia, as there is an overlap in the clinical features across the different dementia types (9–11). There is currently no single marker available that can differentiate between AD and other dementia types. Hence, there is a need for biomarkers that can distinguish between the dementias.

Additionally, successful development of disease-modifying drugs and prevention therapies require biomarkers that can recognize neuropathological changes in the pre-dementia stage and allow differential diagnosis. This would allow inclusion of the right patients in the clinical trials, monitoring of the treatment efficacy, and exclusion of patients that have already reached a point-of-no-return and would not have any beneficial effect of a given intervention (12, 13).

Unfortunately, the biomarker development has been hampered by the fact that tracking molecular pathological changes in the brain is a huge challenge due to the inaccessible nature of the brain. Currently imaging and CSF biomarkers provide the best method for diagnosing, staging, as well as predicting clinical progression of AD and related dementias. However their use is limited by cost, availability and by the fact that repeated brain scans and withdrawal of CSF by lumbar punctures are not advisable (14, 15). These aspects all underline the need for novel biomarkers which are easily obtainable.

## The Proteopathy of Dementia

Most dementias can be designated as proteopathies characterized by aberrant processing of neuronal proteins such as fragmentations, aggregations and other post-translational modifications (PTMs) (Table 1) (3, 16).

**Table 1:** **Common types of dementia and proteins affected**.

Dementia type	Proteins affected	Reference
Alzheimer’s disease (AD)	tau, Aβ, ApoE, α-synuclein	(17–19)
Vascular dementia (VaD)	tau	(20, 21)
Corticobasal degeneration (CBD)	tau	(22)
Dementia with Lewy Bodies (DLB)	α-synuclein	(19)
Parkinson’s disease dementia	α-synuclein	(23)
Frontotemporal lobar dementia (FTLD)		
• FTLD-tau	Tau	(20, 22)
• FTLD-TDP43	TDP43	(24, 25)
• FTLD-FUS	FUS	(26, 27)

The potential of these proteins as diagnostic and prognostic biomarkers has been extensively studied at the protein level. However, these investigations have been limited by the fact that the role of each of these pathological changes throughout the development of dementia is unresolved. This is due to the intrinsic difficulty of detecting the disease before patients display symptoms, which may be 20 years before the earliest cognitive changes are detected (28). Another complicating factor in diagnosing and determining progression of dementia is the significant crossover between the proteins involved in the different types of dementia. Thus, looking solely at protein levels is too simplistic to provide enough detailed information to differentiate between different dementia types. An alternative to this is the application of PTMs as biomarkers for AD. This is not a new approach, since it has already been investigated in the development of CSF-derived AD biomarkers Aβ_1–42_ and phosphorylated tau (p-tau). This presents an excellent example of how understanding the molecular pathology inflicts certain protein fingerprints on key proteins, provides insight not only to central disease mechanisms, but also provides an opportunity to improve the protein’s usage in terms of diagnostic and prognostic value for a specific dementia or even a subtype of dementia.

As we have previously proposed, AD pathology and other dementias may give rise to blood circulating fragments of key neuronal proteins, thereby allowing detection of disease specific post-translationally truncated fragments in the blood (29). This would allow easier and more frequent sampling and analysis and provide earlier diagnosis and prognosis of dementia.

The present review will focus on addressing the potential of disease-specific protein fragmentation for dementia diagnosis and prognosis and how these fragments can be utilized as biomarkers to segregate between the different types of dementia, especially highlighting how the knowledge from CSF protein biomarkers can be applied to investigate blood-based biomarkers.

## Status of CSF Biomarkers

The pathological alterations in the brain at the molecular level are directly reflected in the CSF, therefore this fluid has been the most investigated source for development of biomarkers for AD and related dementias. Aβ_42_, t-tau (total tau), p-tau, and α-synuclein are the most studied CSF biomarkers and their performance has been evaluated in several studies (30). Other biomarkers that will be described in this review are apolipoprotein E (ApoE), TAR DNA-binding protein 43 (TDP-43), fused in Sarcoma protein (FUS), and glial fibrillary acidic protein (GFAP).

Aβ_42_ is the main component in the extracellular amyloid plaques of AD and is a marker of amyloid precursor protein (APP) processing and plaque load. In AD, a decrease in CSF Aβ_42_ has been found, which is probably due to deposition in plaques (17, 31). Generation of Aβ_42_ is an early event in AD, hence measuring CSF Aβ_42_ is a very relevant strategy in prodromal AD to screen for early cases as well as monitoring disease progression. However as it is today the strategy of measuring CSF Aβ_42_ only provides a supplementary test to support the diagnosis once cognitive dysfunction is apparent, and it gives little information on the disease progression as this biomarker has already found a steady-state of abnormality early in the disease progression (32, 33). CSF Aβ_42_ is able to discriminate between AD and non-demented controls with a sensitivity of 59–96% and a specificity of 77–89% (17, 34–36). A change in Aβ_42_ levels has also been studied for other types of dementia and shows a slight decrease in FTLD, DLB, and VaD (32). CSF Aβ_42_ has been shown to predict the rate of cognitive decline in patients with very mild dementia and predict AD in subjects with mild cognitive impairment (MCI) (37, 38).

Cerebrospinal fluid t-tau is a biomarker of neuronal damage and neuronal and axonal degeneration and several studies have shown an increased level in AD patients compared with controls with a sensitivity and specificity of 70–83% and 81–92%, respectively (17, 34–36). However, CSF t-tau is not specific for AD and is also increased in other dementias such as Creutzfeldt–Jakob disease (CJD) patients and in a significant number of patients with DLB, FTLD, VaD, and CBD (20, 32).

Cerebrospinal fluid p-tau reflects aberrant phosphorylation and neurofibrillary tangle (NFT) burden. A strong increase in p-tau has been found in AD using ELISA methods that detect different phosphorylated epitopes such as p-tau(181) or p-tau(231). CSF p-tau differentiates between AD patients and controls with a sensitivity of 68–86% and a specificity of 61–73% (35, 36). A moderate increase in p-tau has also been found in CJD and DLB (17, 20). It has been reported that the use of p-tau instead of t-tau may improve the diagnostic sensitivity and differential diagnosis of AD versus DLB and FTD, respectively (34). Both t-tau and p-tau have been found to predict progression from MCI to AD (32, 39).

The combination of CSF biomarkers (t-tau/Aβ_1–42_ and p-tau/Aβ_1–42_) has been found to increase the sensitivity and specificity when compared to the single markers. The t-tau/Aβ_1–42_ ratio shows a potential as a preclinical biomarker since it discriminates between MCI patients that progress to AD and those that do not progress, although the CSF sampling makes it virtually useless for this purpose (36, 40, 41). Furthermore, the ratio shows promise in prediction of dementia in cognitively normal older individuals (42).

Another interesting CSF biomarker is α-synuclein. Compared to tau and Aβ_1–42_, little research has been done with respect to CSF levels of α-synuclein, which is the main component of Lewy bodies of DLB patients. Studies have demonstrated decreased CSF levels of α-synuclein in DLB and Parkinson’s disease (PD) when compared to controls indicating a potential diagnostic use (43, 44). In contrast to this other research groups have shown no difference in CSF levels in DLB and PD patients compared with controls and other dementias (45–47).

In both PD and DLB patients, the level of α-synuclein oligomers is increased compared to healthy patients and other types of dementias (23, 48). In PD, the ratio of oligomers of α-synuclein to total α-synuclein is also significant. There is an increase in the ratio of oligomeric/total α-synuclein when compared to other dementias (49). Recent studies have also shown significantly elevated CSF levels of α-synuclein in AD patients (50) suggesting that α-synuclein may not be specific to DLB and PD, or again indicating that mixed pathologies are common.

Although, several CSF biomarkers show a promising diagnostic and prognostic potential, there are still important drawbacks limiting their clinical utility (Table 2). An important limitation is the lack of assay standardization and global cut-off values for biomarker concentrations. The handling of CSF and use of different technological platforms and antibodies are the major reasons for significant differences in biomarker concentrations between studies (51). Fortunately, international standardization initiatives have been initiated to reduce the large variations between studies and within laboratories (52). Another limitation of CSF biomarkers is the overlap between the protein profile of different types of dementia (20). Lastly, the clinical utility of CSF biomarkers is still hampered by sample collection, which requires a lumbar puncture. Despite the fact that there is minor complications related to lumbar puncture the procedure is still regarded as invasive in the general population and repeated follow-up measurement is challenging (14, 15), and hence they are not consistently applied in clinical trials. On the other hand, the CSF proteins described here all have a pathological link to the diseases of interest, and as such are of quite some interest for the development of blood-based biomarkers.

**Table 2:** **Advantages and drawbacks of CSF biomarkers**.

Advantages	Drawbacks
Diagnosis	Sampling
Prognosis	Standardization
	Diagnostic cut-off values
	False positive – false negative rates
	Overlap with other dementias

## Status of Blood-Based Biomarkers

The use of blood as a source of dementia biomarkers is still under investigation. Blood is a more feasible biomarker source when compared to CSF due to its wide availability, low cost, time effectiveness, and easier sampling. Several different approaches for identification of blood biomarkers are available and these include biomarkers of the amyloid and tau pathology, biomarkers of inflammation, oxidative stress, mitochondrial dysfunction, neuronal and microvascular injury, and biomarker panels (15, 53). So far, the research has been hampered by two major challenges. The first is the complexity of blood and the large variation in samples and variation between studies. The difference in preanalytical and analytical methods is an important reason for this variation and these have been reviewed elsewhere (15). The second challenge is the fact that blood is not in direct contact with the brain. This limits the understanding of how the pathological alterations in the brain are reflected in blood analytes, as well as the absolute level of the analyte of interest in the blood. Additionally, the prevalent presence of non-specific proteins in the blood is an obstacle toward identification of disease-specific biomarkers. To overcome these limitations, the experience from the well-characterized CSF biomarkers, which in some cases are based on brain-specific pathological alterations, i.e., p-tau, may be a starting point for blood biomarker analysis. Pathological alterations in CSF proteins may be reflected in blood as a consequence of absorption of CSF into blood, by penetration due to barrier impairment in dementia or simply by diffusion (54–58). Whether a brain-derived protein can serve as a biomarker to be measured in blood will depend on the concentration, the change in concentration during disease, the molecular size and the half life in blood (57). Hence, exploring the dynamic range of brain proteins in the peripheral blood is of great interest.

The CSF biomarker tau is a brain-specific protein that can become a relevant biomarker to be measured in blood. So far, little is known about tau levels in blood and most studies have been hindered by the low abundance of the protein in blood (59). Zetterberg et al. (59) found that there was no correlation between CSF tau levels and plasma tau indicating that the clearance of tau is differently regulated (59). In healthy blood-donors tau protein concentration is in the range <10 and >100 pg/mL and the ratio between CSF:serum tau is 10:1 (57). Methods for determining tau in serum/plasma are under investigation. Few studies have reported elevated plasma tau levels in patients with AD (59, 60). The results from these studies are encouraging but highly sensitive detection methods are necessary. An ultrasensitive immunoassay for detection of plasma tau has been introduced and similar methods would be highly relevant (61).

Another CSF biomarker with potential to be a blood biomarker is Aβ. Plasma Aβ species have been examined by numerous studies but the results are contradictory. Some of these studies report high Aβ_42_ or Aβ_40_ whereas others show a decrease in AD. The overlap between patients with AD and healthy controls is also substantial. Importantly, Aβ is not brain-specific but is also expressed by other cells, and as such there is an interference of the peripheral Aβ species with the brain-derived species. Additionally, the binding of Aβ to plasma proteins and formation of Aβ oligomers may disturb the quantification by immunoassays (62, 63).

Finally, several studies have quantified plasma α-synuclein and α-synuclein oligomers in PD and DLB. However, additional studies are needed to evaluate blood α-synuclein as a valid biomarker and the high levels of α-synuclein present in red blood cells must be considered when quantifying the protein (64).

Plasma levels of ApoE, TDP-43, and GFAP have also been reported and the main results from these studies will be reviewed in the next sections.

Altogether, the inconsistent findings from plasma analyses illustrate the need for a pathology specific combination of protein and modification of this protein in order to enhance the possibility of generating a disease-specific biomarker, even more so in blood specimens than CSF.

## Status of Protein Fragmentation Blood-Based Biomarkers

As mentioned identification and detection of brain-specific proteins in blood is restricted by the blood–brain-barrier, the substantial presence of non-specific proteins, and proteins from co-morbidities in the circulation. The use of post-translationally truncated protein fragments containing specific neo-epitopes as biomarkers of dementias may overcome these complexities (29, 65). Targeting protein fragments generated by disease-specific combinations of proteins and proteases opposed to detecting the intact protein could diminish the overlap between diagnostic groups. Proteolytic fragmentation of proteins is a post-translational process and several cleavage products have been identified in relation to AD and other dementias. Aβ_42_, Aβ_40_, and several other N- or C-terminally truncated Aβ peptides all represent examples of proteolytically cleaved protein fragments. Cleavage of tau, ApoE, α-synuclein, TDP-43, and GFAP has also been reported (66–70).

**Figure 1:**
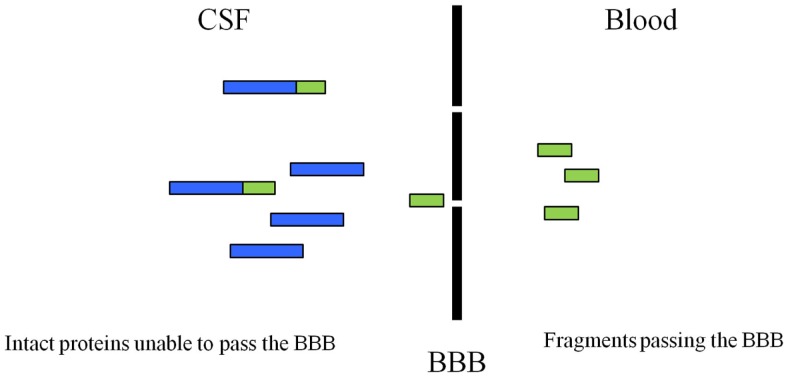
**Illustration of how the protein fragments may be able to cross the blood–brain barrier**. Protein fragments may have the advantage of crossing the barrier as these breakdown products have a smaller size when compared to the intact protein. Modified from Ref. (29).

Although, several of the described protein fragments have been described in the literature and detected in CSF most of these have not been studied in blood. Targeting protein fragmentation by specific proteases may provide novel biomarkers for dementia and create a specific profile of each disorder based on the fragments and proteases that are involved in the pathology. Another advantage of using fragments as blood biomarkers opposed to the intact proteins may be the eased release from the central nervous system (CNS) into the periphery. The fragments may easier pass the blood-brain barrier due to their small size and be easier to detect (71–75) (Figure 1).

In addition to applying disease-specific protein fragmentation to identify new biomarkers for dementia, it is important to define and validate the ability of each novel biomarker. The BIPED classification system (Burden of Disease, Investigative, Prognostic, Efficacy of Intervention and Diagnostic) is a nomenclature first used for osteoarthritis and offers categorization of biomarkers in order to improve the development and validation of biomarkers (15). The use of BIPED classification in dementia would aid in the biomarker development process from target identification to validation in clinical trials.

**Figure 2:**
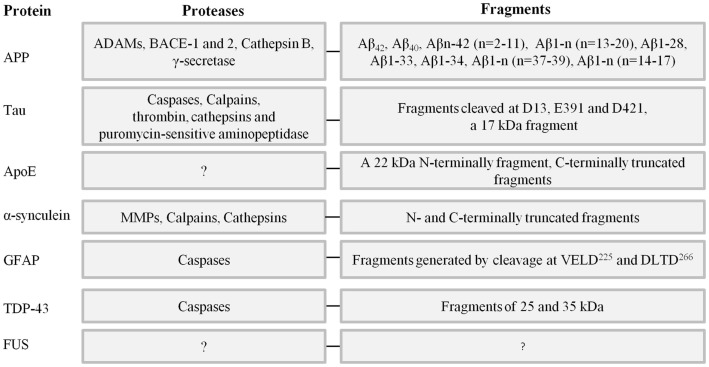
**Key neuronal proteins involved in the proteopathy of different types of dementia, proteases involved in their truncation and fragments known from the literature**. References can be found in the text.

In the following sections, neuronal proteins involved in the proteopathy of dementias will be reviewed with emphasis on proteolytic fragmentations (Figure 2).

## Amyloid Precursor Protein

Derivatives from the full-length APP are the main components of the extracellular amyloid plaques. APPs are type 1 transmembrane proteins and exist in three isoforms in humans, APP695, APP751, and APP770. The APP695 is the main isoform in neurons and is the only isoform containing the sequence encoding Aβ (76, 77). In normal cells, APP is involved in kinase-based signaling, growth regulation, neurite outgrowth, formation of synapses and cell adhesion (33, 78). APP is cleaved by secretases and caspases at specific sites and this leads to the formation and release of several protein fragments (76, 78). The proteolytic processing of APP can follow the amyloidogenic or the non-amyloidogenic pathway. The major component of senile plaques, Aβ, is generated in the amyloidogenic pathway by sequential cleavage of APP by β-secretase and γ-secretase to generate Aβ_40_ and Aβ_42_. BACE1 (β-site APP-cleaving enzyme 1), BACE2 (β-site APP-cleaving enzyme 2), and cathepsin B have been identified as β-secretase responsible for production of Aβ. The γ-secretase activity belongs to a membrane-bound protease complex (presenilin 1, presenilin 2, nicastrin, Aph-1, and Pen-2) (76, 78). In the non-amyloidogenic processing, APP is cleaved by α-secretase which binds to and cleaves APP within the Aβ region and prevents formation of Aβ. All the identified α-secretases are from the family of disintegrin and metalloproteases (ADAMs).

The accumulation of Aβ is an early process in neurodegeneration leading to formation of oligomers, fibrils, and eventually extracellular plaques. CSF Aβ_42_ levels become abnormal 5–10 years or more before the diagnosis (79, 80). The concentration of CSF Aβ_42_ begins to increase abnormally followed by a drastic decrease. In mutation carriers (i.e., in the APP genes, presenilin 1, or presenilin 2), CSF Aβ_42_ levels become abnormal up to 25 years before disease onset (28). Intracellular levels of Aβ initiate synaptic dysfunction, formation of NFTs and loss of neurons. The Aβ_42_ is the main toxic form of Aβ, whereas Aβ_40_ has been shown to have neuroprotective functions (78, 81).

Aβ_42_ and Aβ_40_ have also been detected in patients with cerebral amyloid angiopathy (CAA), which can be a co-occurring disorder with AD or a separate finding. CSF levels of Aβ_42_ and Aβ_40_ are lower in patients with CAA and CAA-related inflammation (CAA-ri) than controls (82–84). Furthermore, the level of CSF anti-Aβ autoantibodies is increased in CAA-ri which shares similarities with the amyloid-related imaging abnormalities detected in AD immunization clinical trials (84). It has been suggested that the CSF anti-Aβ autoantibody concentration can be used as a biomarker during immunization clinical trials in AD (84, 85).

The Aβ peptide is subjected to further truncations by different proteases and forms peptides of various lengths. The peptides are generated by N- or C-terminal truncation of Aβ and several of these have been identified in CSF, e.g., Aβ*_n_*_–42_ (*n* = 2–11), Aβ_1–_*_n_* (*n* = 13–20), Aβ_1–28_, Aβ_1–33_, Aβ_1–34_, and Aβ_1–_*_n_* (*n* = 37–39). These peptides have been found to be elevated in CSF of AD patients but only few are involved in plaque formation (86–89).

Recently, it was reported that some of the identified Aβ peptides in CSF are generated by an alternative APP processing pathway (90). In this pathway, APP is cleaved by α- and β-secretase without the involvement of γ-secretase. Many of the peptides derived from this pathway are elevated in CSF from AD suggesting an up-regulation of this pathway in AD as a response to the increase of the amyloidogenic pathway (90). The identified products of the alternative pathway are Aβ_1-14_, Aβ_1-15_, and Aβ_1-16_. Eleven other truncated peptides with C-terminal at residue 15 in the Aβ sequence and start at the N-terminal end of the β-secretase site have been identified in CSF. The peptides contain a part of the Aβ sequence but are not degradation products of Aβ because they start upstream of the β-secretase cleavage site. Several of these were found to be elevated in AD and may also be generated in the alternative processing pathway (91).

Plasma levels of Aβ_42_, Aβ_40_, and the ratio Aβ_42_/Aβ_40_ have been examined in several cross-sectional studies with AD, MCI patients, and healthy controls. The results have shown a substantial overlap between diagnostic groups and the results between studies have been contradictory (92). Aβ_42_ and Aβ_40_ have also been studied in longitudinal studies to assess their association with disease progression. Although the results are not clear between individual studies the data show that a decreased baseline level of Aβ_42_ predicts a greater risk of AD (92). A recent study has quantified Aβ_1-17_ levels in plasma and has shown significant associations with the clinical diagnosis of AD, indicating the potential of the Aβ fragments (93). The plasma levels of the remaining Aβ cleavage products have only been examined in few studies. Highly specific antibodies and robust immunoassays must be developed and used for detection of these cleavage products of different size.

## Tau

Tau is the basic component of the intracellular insoluble filamentous structures, also referred to as NFTs. The tau protein belongs to the family of microtubule-associated proteins and binds to, stabilizes, and promotes the assembly of microtubules. Tau is also involved in signaling pathways and cytoskeletal organization (94).

Tau is mainly expressed in the central and peripheral nervous system and most abundant in axons. There are six isoforms in the adult human brain, which vary in size and have either three or four microtubules-binding domains. The six forms each show functional differences (95, 96). The ratio between tau containing three and four domains is 1:1 in normal human brain but this ratio is altered in the different tauopathies. Additionally, different isoforms of tau are involved in the different tauopathies and affect distinct brain regions, hence it has been suggested that the isoform profiles can be used to classify the different tauopathies (97, 98). Besides AD, the tauopathies include FTLD, progressive supranuclear palsy (PSP), CBD, and prion diseases (20, 98).

In AD, the concentration of CSF t-tau and p-tau become abnormal after Aβ_42_ and their levels increase progressively up to the time of diagnosis. Thus, tau levels are higher in MCI patients with an early conversion compared with late converters (79, 80). Increased CSF levels of tau are increased 15 years before symptoms in mutation carriers (28).

The conversion of soluble tau protein to insoluble inclusions is a central event in AD and other tauopathies. Formation of inclusions is mediated by protein aggregation and misfolding. The aggregates have been shown to be self-propagating and spread from one neuron to another (99). Tau aggregation and misfolding are induced by abnormal phosphorylation and proteolytic cleavage. Hyperphosphorylated tau is the main component of NFTs and several kinases and phosphatases have been associated with this. A level of phosphorylation occurs at normal state but in disease state, an abnormal level of phosphorylation is seen and results in a low-binding affinity to tubulin promoting disassembly of microtubules (94, 96).

Although the presence of t-tau and p-tau in CSF has been investigated in several studies, the nature of the protein in CSF is not fully known. A number of studies have suggested the presence of different tau and p-tau fragments in CSF (94, 95) and a recent study has reported that CSF tau and p-tau occur as various N-terminal and mid-domain fragments (67). The level of specific fragments were significantly elevated in AD patients when compared to controls and showed a diagnostic potential but the fragments still remain to be measured in other dementias (67).

Plasma levels of t-tau and tau fragments have only been assessed in few studies. It has been demonstrated that plasma t-tau levels are elevated in AD patients but with an overlap with control subjects (59). Hence, the diagnostic utility of plasma t-tau is not clear. Recently, the presence of protease generated fragments of tau has been shown in serum (75, 100, 101). The fragments have been shown to correlate with symptoms in AD patients and predict the disease progression in early AD (100, 101), indicating the pathological relevance of fragmentations.

It is a possibility that the assays for t-tau may also detect certain fragments of tau and as multiple systems are in use for detecting t-tau, this is most likely different from assay to assay depending on the antibodies used. Unless an assay is constructed as a sandwich ELISA with antibodies detecting the N- and C-terminal sequences, there is this possibility.

Furthermore, it must be noticed that the relative concentration of the protein determined in the clinical studies is a result of the specific calibrators used in the different assays.

In dementia, tau is cleaved by caspases and calpains, but other proteases have also been detected including thrombin, cathepsins, and puromycin-sensitive aminopeptidase (102). It has been found that certain proteolytic fragments of tau are specific for the different tauopathies suggesting that different proteases may be specific to individual tauopathies (102). Several tau fragments have been reported and the most studied are caspase-generated tau fragments cleaved at D13, E391, and D421 as well as a calpain-cleaved fragment of 17 kDa which are associated with AD (66, 103). The majority of the reported fragments have only been analyzed *in vitro*, in AD-affected brains or transgenic animals (94).

## Apolipoprotein E

The ε4 allele of ApoE is known to be associated with the risk of developing AD. ApoE is a major transport protein of cholesterols and other lipids in plasma and in the brain. It is most abundant in the brain and the liver (104). In the CNS, ApoE is mainly synthesized in astrocytes but is also present in lower concentration in some neurons, activated microglia, oligodendrocytes, and ependymal layer cells. In neurons, the synthesis of ApoE is induced under neuronal stress and damage and has been detected in cortical and hippocampal neurons (105). In the normal brain, ApoE is associated with the maintenance and repair of neurons and involved in the cholesterol homeostasis (106). ApoE is a polymorphic protein with the main isoforms being ε2, ε3, and ε4. The three isoforms differ by single amino acid substitutions at positions 112 and 158 (104, 107). The ApoE ε4 allele is a risk factor for late-onset familial and sporadic AD (18, 108). Around 10–15% of the general population has the ε4 allele, whereas the prevalence is 40–65% in AD patients. The majority of the general population is homozygous for the ApoE ε3 allele. The third common isoforms ε2 is present in 5–10% of the population. The ApoE ε2 allele has protective effects on the cognition and has been associated with reduced AD-related disease burden (109, 110).

Homozygosity for ApoE ε4 leads to a 50–90% risk of developing AD by the age 85, whereas individuals with one copy have a risk of 45%. For individuals with no ApoE ε4 alleles the risk is about 20% (18, 111). ApoE has been found to be co-localized with amyloid plaques and NFTs (105). Several mechanisms have been proposed for the role of ApoE ε4 in the pathology of AD including regulation of the deposition and clearance of Aβ and amyloid plaques, regulation of phosphorylation and assembly of tau into NFTs, dysfunction of the neuronal signaling pathways, induction of Aβ-regulated lysosomal leakage, increased atherosclerosis and vascular inflammation in AD, and apoptosis in neurons (105, 112). However, its exact role in the AD pathology still remains unclear (105). Besides AD, the ε4 allele has also been associated with CAA, hemorrhages, tauopathies, DLB, PD, and multiple sclerosis (113–116).

The CSF, ApoE levels have been determined by several studies and some have found decreased levels in CSF of AD patients whereas other studies have shown an increase (117). Increased CSF levels of ApoE were also detected in DLB and PD patients (118).

Plasma ApoE levels have also been reported but as seen with the CSF measurements the results have been inconsistent. A study by Taddei et al. (119) reported increased plasma ApoE levels in AD patients compared to controls. In contrast to this, the Australian Imaging, Biomarkers and Lifestyle (AIBL) study showed decreased plasma levels of ApoE and ApoE ε4 in AD patients and showed a correlation with the disease level (120). Two other studies based on the Rotterdam study and apoEurope Study, respectively, also observed decreased ApoE levels in AD patients compared to controls (121, 122). However, this difference was not significant in the Rotterdam study when adjusted for ApoE genotype, age, and gender (121). Finally, a recent study has shown that low plasma ApoE levels are associated with the risk of developing AD independent of the ApoE genotype, indicating the potential of this biomarker as a preclinical marker (123).

Aberrant proteolytic cleavage of ApoE plays an important role in the AD pathology associated with ApoE. ApoE is subjected to intracellular proteolytic cleavage and generates neurotoxic fragments. The fragments have been detected in cultured neurons and AD brains and have been shown to induce tau phosphorylation and formation of NFT-like aggregates in CNS neurons with p-tau and phosphorylated neurofilaments (124, 125). In addition, the fragments impair the function of mitochondria in neurons and promote neurodegeneration. The level of ApoE fragments is elevated in AD brains compared to cognitively normal controls (68). Importantly, ApoE ε4 is more susceptible to fragmentation than ApoE ε3 (124, 126). Among the fragments, a 22 kDa N-terminally peptide has been detected in brain tissue and CSF. Interestingly, the ApoE ε4-derived 22 kDa fragment has been found to be more neurotoxic than the corresponding ApoE ε3-derived fragment (68). Several C-terminally truncated ApoE fragments of different lengths have also been detected in AD brains. One of these is the apoE4 (Δ272–299) fragment which interacts with p-tau and phosphorylated neurofilament to form inclusions (124). A neuro-specific chymotrypsin like protease has been suggested to be involved in the formation of these fragments but further studies are needed (127).

So far, there are no studies on plasma levels of ApoE fragments and their correlation with AD or other dementias.

## α-Synuclein

α-synuclein is a small protein located in both the CNS and the peripheral nervous system. It can be found specifically bound to the membrane of pre-synaptic vesicles and very little α-synuclein is distributed throughout the rest of the nerve (128). α-synuclein is also expressed in other tissues including red blood cells (64), kidney, lung, heart, and liver (129). The specific function of α-synuclein is unknown but it is implicated in a number of dementias including AD, DLB, and PD. α-synuclein aggregates to form a component of Lewy bodies that can be found in the cytoplasm of neurons. These aggregates are observed in the dementias mentioned above except for AD and are believed to be the key step in progression of neurdegeneration in synucleionopathies. There is, however, evidence that suggests α-synuclein plays a role in the aggregation of tau, which is observed in AD (130, 131). Furthermore, increased levels of soluble α-synuclein have been found in AD brains in patients in absence of LBD pathology and the levels showed a correlation with cognitive decline (132).

Cerebrospinal fluid levels of α-synuclein and its oligomers have been assessed in several types of dementia. The differential performance of α-synuclein has been inconsistent in different clinical studies. A number of studies have shown that CSF α-synuclein levels are lower in DLB and PD patients than those with AD and other dementias (43, 44, 133), whereas others have concluded that CSF α-synuclein does not discriminate between dementias (46). The levels of CSF α-synuclein oligomers are increased in DLB and PD compared with controls and AD patients (48).

The plasma levels of α-synuclein and its oligomers have been quantified in DLB and PD patients by several studies. Increased plasma levels of α-synuclein and oligomers were seen in patients with PD when compared to controls (134–137). However, contradictory results were observed in other investigations (138, 139). Similarly, the level of plasma α-synuclein oligomers was higher in DLB patients than controls whereas the α-synuclein levels were lower in DLB than AD patients and controls (134, 139).

A lot of focus has been on aggregation of the intact α-synuclein, however more recently studies suggest that fragmentation of α-synuclein is significant in the pathology of synucleinopathies. Fragments of α-synuclein have been identified in brains of PD and DLB patients (69, 141). One protease of interest is calpain, which has been observed to create cleavage products that can induce aggregation of α-synuclein *in vitro*. Calpain cleaves α-synuclein in the N- and C-terminal regions (140). MMPs also play a role in α-synuclein aggregation and therefore Lewy Body formation. Partial cleavage with either MMP-1 or MMP-3 increases aggregation of the protein (141) and both proteases are elevated in PD brains (142, 143). Neurosin is another protease of interest, especially as it is found within amyloid plaques in AD (144). Neurosin has also been identified in CSF and has been found to be lower in patients with synucleinopathies compared to those with AD and healthy patients (145). Finally, cathepsins are known to be involved in the proteolysis of α-synuclein (146). The presence of α-synuclein fragments in CSF and plasma remains to be investigated.

## TAR DNA-Binding Protein 43 and Fused in Sarcoma Protein

TAR DNA-binding protein 43 is a nuclear protein that functions in regulation of transcription and exon splicing (24, 147). TDP-43 is known as the key protein in the pathogenesis of FTLD with ubiquitin-positive, tau-negative inclusions. FTLD is the second most common type of dementia after AD with an onset before 65 years of age (148) and differentiation between AD and FTLD can be challenging as they share several clinical features (149).

In FTLD, TDP-43 is post-translationally modified by aberrant ubiquitination, hyperphosphorylation, and proteolytic cleavage at the N-terminus (24, 25). In addition, TDP-43 is translocated from the nucleus and generates cytoplasmic insoluble inclusions containing ubiquitinated and aberrantly phosphorylated TDP-43 (24).

TAR DNA-binding protein 43 neuronal and glial inclusions have been detected in AD and several types of PD (150). TDP-43 inclusions are found in 25–30% of all sporadic AD patients and 14% of familial AD patients. The presence of TDP-43 in AD brains has been shown to give greater brain atrophy and more deficits when compared to AD patients without TDP-43 inclusions (151). In addition, caspase 3-cleaved TDP-43 has been detected in AD brains and it is proposed to be associated with neurodegeneration (70). This suggests that TDP-43 in combination with specific AD biomarkers can be used to identify patients with the risk to develop severe clinical deficits.

TAR DNA-binding protein 43 levels are detectable in CSF and were found to be elevated in FTLD patients when compared to controls (152, 153). TDP-43 has also been detected in plasma and the levels were increased in FTLD and a subset of AD patients (154, 155).

Fragmentation of TDP-43 has been observed. The N-terminal cleavage of TDP-43 generates C-terminal fragments, but the cleavage sites and their function in the pathology of FTLD are not fully known. In an *in vitro* study, two caspase-generated C-terminal fragments of 25 and 35 kDa were identified (156). The 25 kDa fragment of TDP-43 was found to induce the formation of intra-cellular toxic, insoluble and ubiquitin- and phospho-positive aggregations. Hence, protease cleavage initiates the translocation of TDP-43 from the nucleus to cytoplasm and induces formation of toxic insoluble inclusions (25). Caspase 3, 7, 6, and 8 have all been associated with TDP-43 cleavage (156).

The TDP-43 fragments have not been investigated in CSF or plasma.

TAR DNA-binding protein 43 and its fragments are potential biomarkers for tau-negative FTLD and can be used in the differential diagnosis of dementia and aid in the separation between tau-negative FTLD and tauopathies.

Another protein with implication for the differential diagnosis of dementia is the RNA-binding protein fused in sarcoma. The FUS protein is the pathological protein in 10–20% of sporadic FTLD patients (FTLD-FUS), which are negative for TDP-43 (26, 27, 157). The FUS protein binds to DNA and RNA and is associated with several cellular processes such as cell proliferation, DNA repair, transcription regulation, RNA splicing and transport of RNA (158–162). FUS is ubiquitously expressed in the nucleus and cytoplasm in most cell types and in neurons and glial cells it is primarily expressed in the nucleus (163). In FTLD, the FUS protein is mostly present in the cytoplasm whereas the FUS levels in the nucleus are decreased indicating a delocalization of the protein. The delocalization and accumulation of FUS lead to formation of cytoplasmic inclusions that are the characteristics of FTLD-FUS (26, 150). In addition, a mouse model has shown that overexpression of the FUS protein results in neurodegeneration (164).

To the best our knowledge neither the levels of FUS in CSF and plasma nor its fragmentation have been reported.

## Glial Fibrillary Acidic Protein

Glial Fibrillary Acidic Protein is a type III intermediate filament (IF) protein constituting a part of the cytoskeleton in specific cell types. Besides the pivotal role of GFAP in the structural properties of these cells, it is involved in several fundamental cellular activities including motility (165), autophagy (166), synapse formation (167), and myelination (168).

Although it was originally considered an astrocyte-specific marker (169), GFAP has subsequently been demonstrated in glial and non-glial cells of the periphery (170–173). GFAP has been observed in virtually all areas of the brain but is mainly expressed in hippocampal regions (174–176) as well as the subventricular zone and olfactory system of both non-demented elders and patients with dementia (174–177). Multiple splice variants exist and in human hippocampal AD tissue many of these isoforms show differential transcript levels (176).

Differential transcript levels of GFAP isoforms may affect cellular function and/or morphology (165) as analysis of *in vitro* transfection suggests that GFAP isoforms differ in their ability to form functioning IFs (174, 176, 178, 179). In general, little is known about the role of GFAP in AD and other dementias. GFAP is known to interact with proteins involved in cleavage of APP (180, 181) as well as proteins modulating chaperone mediated autophagy (CMA) (166). GFAP may both inhibit and promote CMA and the phosphorylation state of GFAP is suggested to influence this balance (166). Incomplete CMA of tau is suggested to promote tau aggregation (182) which is a hallmark of several tauopathies including AD (103).

Studies have shown a correlation between increased expression levels of GFAP within brain regions involved in memory and the neuropathological changes of AD such as Aβ deposits and NFTs (183–187). Also, disease duration and progression of AD has been shown to correlate strongly with up-regulation of GFAP in the temporal lobe of AD patients (176, 184, 188).

In CSF, levels of GFAP have been observed to be increased in AD patients compared to controls (189–192). Furthermore, GFAP levels were found to increase with AD severity (189). In most studies, increased GFAP levels were independent of age, however, Rosengren et al. (190), observed a correlation between these two parameters (190).

Cerebrospinal fluid GFAP levels are also increased in patients with other neurological disorders and brain injuries such as CJD (191, 192), stroke (193, 194), and traumatic brain injury (195, 196). Regardless of this general increase in GFAP levels observed in these disorders and injuries, GFAP may be applied in context with other biomarkers for differential diagnosis, e.g., GFAP, together with the glial-specific S100 calcium binding protein B (S100β) may hold the potential to distinguish between CJD and AD (191).

In a recent study, GFAP was measured in plasma. Patients covering a broad spectrum of neurological diseases, including several forms of dementia, were included. Plasma levels of GFAP were found to be independent of age and evenly distributed between genders. No disease category displayed consistently increased levels of GFAP (197).

*In vitro*, GFAP is cleaved by caspase 6 at VELD225. The result is a C-terminal fragment of GFAP unable to assemble into filaments and an N-terminal fragment of GFAP perturbing *in vitro* filament assembling and promoting inter-filament aggregation (198). Caspase 3 is suggested to cleave GFAP at DLTD266. Cleaved GFAP has been shown to co-localize with caspase 3 in apoptotic astrocytes around blood vessels as well as plaque-rich regions of specific areas in the human AD brain (199). Furthermore, studies have shown calpain I-mediated cleavage products of GFAP in human brain as well as in CSF following traumatic brain injury (200, 201). Taken together, these data suggest that GFAP is a target of calpain I, caspase 3, and caspase 6 and that astrocyte injury and damage in the AD brain may involve cleavage of GFAP.

## Conclusive Remarks

In the last decades several biomarker candidates have been developed and evaluated for AD and related dementias. Given the multiplicity of proteins involved in AD and related dementias as well as the overlap in pathological features between the different dementias it has to be acknowledged that so far no single biomarker permits an accurate and differential diagnosis. The diagnostic performance of the identified biomarkers could be improved by focusing on the pathological fragmentation of these proteins.

Although further studies are needed to evaluate the performance of protein fragmentation biomarkers, we believe that these biomarkers either alone or in combination with other biomarkers have a clinical potential.

## Conflict of Interest Statement

All authors are employed by Nordic Bioscience Biomarkers and Research. Kim Henriksen and Morten Asser Karsdal hold patents on biomarkers of neurodegeneration. Morten Asser Karsdal holds stock in Nordic Bioscience.
